# From evidence to action: expert perspectives on stakeholder dialogues in Swiss health policy

**DOI:** 10.3389/ijph.2026.1609991

**Published:** 2026-09-18

**Authors:** Sara Rubinelli, Cristina Mesa-Vieira, Isabelle Peytremann-Bridevaux, Nicola Diviani, Sarah Mantwill, Natalie Gray Messerli, Maddalena Fiordelli

**Affiliations:** 1 Faculty of Health Sciences and Medicine, Universitat Luzern, Lucerne, Switzerland; 2 Department of Epidemiology and Health Systems, Unisanté, University Center for Primary Care and Public Health and Université de Lausanne, Lausanne, Switzerland; 3 Schweizer Paraplegiker-Forschung, Nottwil, Switzerland; 4 Institute of Public Health, Faculty of Biomedical Sciences, Università della Svizzera Italiana, Lugano, Switzerland

**Keywords:** deliberative processes, evidence-informed policymaking, health policy, knowledge translation, qualitative research

## Abstract

**Objectives:**

Stakeholder dialogues are increasingly used to link research evidence with policy and practice in health systems. Yet little is known about the practical reasoning behind their design, conduct, and use. This study investigated how researchers and public-health professionals conceptualise and operationalise stakeholder dialogues, with a primary focus on Switzerland’s federal, multilingual health system and broader relevance to other settings.

**Methods:**

We conducted semi-structured interviews with fourteen experts involved in designing, facilitating, or implementing stakeholder dialogues in Switzerland and internationally. Interview data were analysed using reflexive thematic analysis.

**Results:**

Stakeholder dialogues emerged as structured, evidence-informed discussions linking research to implementation. Success depended on high-quality evidence briefs, skilled neutral facilitation, and inclusive formats enabling equal participation. Consensus was not required; clarified disagreement and transparent reasoning were legitimate outcomes. Impact ranged from mutual learning to policy uptake, shaped by federalism and linguistic diversity.

**Conclusion:**

Stakeholder dialogues function as boundary infrastructures that integrate evidence, experience, and values. When deliberately designed, they enhance the quality of reasoning, trust, and coordination, transforming evidence use from a technical exercise into a relational practice in health policymaking.

## Introduction

Policymaking in health is an inherently complex, value-laden enterprise shaped by political institutions, competing interests, and social norms [1–3]. Research evidence alone rarely settles policy questions: it may be misaligned with context, difficult to access, or challenging to translate into implementable steps [4, 5]. The relationship between evidence and policy is further complicated by the political nature of health decisions, where multiple social norms and value systems intersect [6–8]. Experts increasingly advocate for approaches that combine the best available evidence with deliberation among those who will enact and live with policy decisions [5–9].

Within this knowledge-translation agenda, stakeholder dialogues have emerged as a key strategy for integrating synthesised evidence with the values, situated knowledge, and operational constraints of actors across the health system [10]. The “deliberative turn” in democratic theory [11], that is, the shift from treating legitimate collective decisions as the outcome of voting and bargaining alone to grounding them in public reasoning among those affected, has profoundly influenced health policy with deliberative processes now viewed as mechanisms for achieving legitimacy through transparent reasoning among affected parties [11, 12]. While stakeholder dialogues are often associated with agenda-setting and option appraisal, their use now extends across the policy cycle, including later stages of implementation where front-line feasibility and organisational constraints become prominent [10, 13–15]. By front-line feasibility we mean whether those who deliver services can actually enact a recommended course of action under real-world staffing, funding, and organisational conditions. For example, one dialogue described in this study was convened because clinical guidelines were failing to take hold in primary-care practice owing to time and resource constraints on those delivering care; stakeholders worked through what implementation would require of front-line professionals and which adaptations were feasible (see Results below). Dialogues are also increasingly used in practice settings, such as guideline development within professional associations, clinical networks, or organisational units, where decision-making dynamics, evidence needs, and expected outputs differ from those of formal policy processes [16, 17]. Across these contexts, dialogues provide structured, evidence-informed spaces oriented toward action: clarifying options, surfacing trade-offs, and identifying next steps where possible [10, 18, 19].

Evidence shows that stakeholder dialogues have convened community, organisational, and system leaders around pressing public-health challenges; assessed which policy or practice options are likely to work in specific contexts; and presented knowledge in forms usable by decision-makers [18, 19]. In the European context, the CHRODIS PLUS joint action demonstrated how national policy dialogues across fourteen EU member states catalysed cross-sectoral deliberation on chronic disease prevention and management [15]. In Switzerland, the primary setting of this study, stakeholder dialogues have likewise been used to connect research with policy and practice: the Swiss Learning Health System, for instance, convenes researchers, policymakers, and practitioners in recurrent stakeholder dialogues built around evidence briefs to address problems of health-system performance [20], and the permanent Dialogue on National Health Policy offers an institutional platform through which the Confederation and the cantons coordinate health-policy priorities [21]. Beyond tangible outputs (e.g., option lists, recommendations, or briefs), dialogues can generate subtler effects, including heightened problem awareness, shared understanding of the evidence base, and reframing of how issues are approached [4, 13].

Effective dialogue, however, is not automatic: the literature highlights enabling conditions such as openness to divergent views, sufficient time, staffing, and resources, transparent processes, clear and accessible evidence inputs, and skilled facilitation adapted to group dynamics [1, 9, 22]. Abelson and colleagues [23] show that design features—representativeness, information provision, rules of interaction—strongly influence outcomes and perceived legitimacy; design must therefore be adapted to problem and setting rather than applied as a template.

Despite growing uptake, conceptual and practical gaps remain. Terms such as “stakeholder,” “participation,” and “community” are used inconsistently, and debates persist over who should be included and on what representational basis [10]. A recent scoping review found that researchers’ involvement in health policy dialogues varies widely, underscoring the heterogeneity of dialogue practices [24]. Equity considerations, management of conflicts of interest, and guidance on which design features suit particular problems and contexts remain underdeveloped [4–6]. Expectations about outcomes also differ: some authors deem consensus unnecessary, emphasising reason-giving and clarified disagreement, while others expect dialogues to generate bounded agreements that support implementation [10, 23, 25].

This study contributes to these debates by examining how experienced researchers and public-health practitioners conceptualise and use stakeholder dialogues across policymaking, implementation, and practice settings, with a particular focus on Switzerland’s multilingual federal system. We explore their views on the purpose and value of dialogues, key design features—including evidence inputs, facilitation, and format—and the barriers and facilitators encountered in practice. The analysis aims to support more intentional and context-sensitive design of stakeholder dialogues as bridges from evidence to action.

## Methods

### Study design and setting

We carried out an exploratory qualitative study using semi-structured interviews with experts who design, facilitate, or regularly participate in stakeholder dialogues in health policymaking and practice settings such as organisational decision-making, service planning (i.e., decisions about how services such as prevention programmes, rehabilitation, or community nursing are organised, staffed, and funded), and guideline development. The dialogues our participants convene or use occur at different points in the policy process, from agenda-setting and the appraisal of options through to implementation and evaluation, and span settings ranging from federal and cantonal public-health administration to hospitals, rehabilitation and long-term care providers, professional associations, and non-governmental organisations. Although the study focused on the Swiss health system, a small number of international experts were included to provide comparative context and to identify aspects of dialogue design that may transcend national borders. This sampling approach allowed us to analyse how stakeholder dialogues are understood and implemented across settings with different institutional logics and constraints. Because the Swiss setting frames our findings, a brief description is warranted. Switzerland is a federal state in which responsibility for health is shared between the Confederation and 26 cantons, the member states of the federation. Cantons hold primary responsibility for securing healthcare provision, including hospital planning, the licensing of professionals, and prevention activities, while the federal level regulates the mandatory health insurance that finances care delivered by a mix of public and private providers [21]. Health policymaking is therefore highly decentralised and consensus-oriented: major reforms typically require agreement among the Confederation, the cantons, and corporatist actors such as insurers, professional associations, and provider organisations, and most decisions can ultimately be challenged by popular referendum. The stakes of failing to reach mutual understanding are considerable (reforms that lack broad stakeholder support are routinely delayed, diluted, or rejected at the ballot box) and coordination is further complicated by the country’s four national languages. In such a system, structured stakeholder dialogue is not merely desirable but often a practical precondition for policy change [10, 21].

### Sampling and participants

We used purposive sampling, complemented by snowball sampling, to identify participants with substantial experience convening or using stakeholder dialogues. Potential participants were identified through the professional networks of the research team, the network of the Swiss School of Public Health (SSPH+), and authorship of relevant publications and project reports, and were approached individually by email invitation. To be eligible, individuals had to have extensive involvement in organising, designing, conducting, or moderating stakeholder dialogues, or in using their outputs for decision-making, not merely attending a dialogue as a participant. Throughout this article, the terms “expert” and “experienced researcher” refer to individuals who met these eligibility criteria, that is, researchers or public-health professionals with substantial, hands-on responsibility for organising, designing, moderating, or using stakeholder dialogues, rather than to academic seniority alone.

### Data collection

Interviews lasted 30–60 min and were scheduled at participants’ convenience. All participants received written information about the study in advance and provided informed oral consent before the interview; consent covered participation, audio-recording, and the use of de-identified quotations. Ten interviews were conducted via Zoom, two in person, and two participants provided written responses due to time constraints. Online interviews were audio-recorded directly in Zoom; immediately after each session, recordings were transferred to secure, access-restricted institutional storage and deleted from the platform. All verbal interviews were conducted in English, audio-recorded with consent, and accompanied by simultaneous field notes; written responses were treated as transcripts and entered into the same analytic workflow.

The interview guide (Supplementary Table S1) covered: definition and perceived value of stakeholder dialogue; formats and facilitation; contrasts with other methods; planning and preparation; documentation and reporting; impact and feedback; roles and responsibilities; engagement strategies; conflict and consensus building; barriers; and facilitators and best practices. Questions were provided to participants in advance. No incentives were offered. Audio files were transcribed verbatim and de-identified; institutional affiliations and potentially identifying details were removed. Written responses were analysed alongside interview transcripts and treated as equivalent data sources. All materials were stored on secure drives accessible only to the research team.

### Analytic approach

We analysed the data using reflexive thematic analysis [26]. This is an interpretive approach in which themes are actively constructed by the researchers as patterns of shared meaning across the dataset, and in which researcher subjectivity is treated as an analytic resource to be examined reflexively rather than as a bias to be eliminated [26]. We considered it well suited to our aim, i.e., mapping how experts conceptualise, design, and use dialogue processes, because our interest lay in practice-oriented reasoning and organisational processes rather than in the lived, phenomenological experience of individual participants. Within this approach, we applied a hybrid deductive–inductive strategy. Two members of the research team independently open-coded an initial subset of transcripts using sensitizing domains from the interview guide (e.g., definitions, design and format, facilitation, barriers and facilitators, impact), while remaining attentive to emergent concepts. Coding was collaborative and interpretive, supported by a flexible and evolving coding framework.

The dual role of two authors as both participant and researcher was acknowledged from the outset. To mitigate potential bias, these individuals did not code or analyse their own transcripts, and interpretation was developed collaboratively across the wider team. Their inclusion was justified by their substantive expertise in stakeholder dialogues and ensured transparency regarding their dual role. All themes were reviewed by team members not involved in that interview.

### Sampling adequacy and saturation

We judged adequacy using an information-power rationale [27] (based on aim specificity, sample specificity, quality of dialogue, and analytic strategy) rather than a numeric target. Given our focused aim, an experienced and specific sample, and interviews of 30–60 min, fourteen experts were sufficient. We monitored theme development through codebook versioning and a framework matrix; by approximately interviews 9–10 the thematic architecture had stabilised, with remaining interviews elaborating and nuancing extant themes rather than generating new ones. We therefore report thematic sufficiency rather than claiming theoretical saturation.

Transferability was addressed through detailed description of the Swiss policy context (federalism, multilingual practice, stakeholder ecology) and by explicitly flagging where examples were context-specific versus broadly applicable. Reporting follows the COREQ 32-item checklist [28]; a completed checklist is provided as Supplementary Table S2.

## Results

Fourteen experts participated: eleven affiliated with Swiss institutions and three with international institutions (Table 1). The Swiss-weighted composition ensured depth and contextual specificity within the national health system, while the three international experts (selected for methodological and policy expertise) provided analytic contrast, allowing us to probe the plausibility and boundary conditions of themes beyond Switzerland. Participants were predominantly university professors and senior managers of research institutions operating across academic, public, and professional or advocacy sectors.

**TABLE 1:** Participant characteristics (Switzerland, 2024–2025).

#	ID	Role	Institution type
1	P01	Researcher/research manager	Health organisation
2	P02	Researcher/research manager	Research institution
3	P03	Researcher/research manager	Health organisation
4	P04	Senior academic	Research institution
5	P05	Senior academic	University
6	P06	Senior academic	Policy/other organisation
7	P07	Expert practitioner	Health organisation
8	P08	Expert practitioner	University
9	P09	Researcher/research manager	Health organisation
10	P10	Senior academic	University
11	P11	Expert practitioner	Policy/other organisation
12	P12	Researcher/research manager	University
13	P13	Expert practitioner	Policy/other organisation
14	P14	Expert practitioner	Health organisation

Role categories: Researcher/research manager = mid- to senior-level researcher leading or coordinating research projects on stakeholder dialogues; Senior academic = full professor or equivalent with a strategic and scholarly role in deliberative methods; Expert practitioner = senior professional who designs, facilitates, or commissions dialogues in policy, advocacy, or health-service settings.

Across the fourteen interviews, stakeholder dialogue emerged as a distinct, practice-oriented form of deliberation that sits between research and implementation. Below we synthesise six cross-cutting themes, integrating illustrative quotations (de-identified by participant ID). An overview of the main themes and subthemes is presented in Table 2. Table 3 lists the quotes that illustrate the various themes presented below.

**TABLE 2:** Main themes and subthemes derived from the reflexive thematic analysis (Switzerland, 2024–2025).

Main theme	Subthemes/dimensions
What “counts” as a stakeholder dialogue	Distinction from data-gathering methods; focus on deliberation and action; value of clarified disagreement
Design levers that make (or break) deliberation	Evidence inputs (policy briefs, preparatory notes); agenda design and moderation; format and participation methods
Power, equity, and legitimacy	Stakeholder mapping (interest × influence); representation and inclusiveness; managing conflicts of interest
From talk to traction	Tangible outputs and commitments; follow-up mechanisms (reports, Delphi rounds); accountability and learning
What impact looks like	Multi-level and delayed effects; hard versus soft indicators; clarified disagreement as systemic learning
“Swissness” matters	Federal and multilingual context; scale and small-network dynamics; practical adaptations

**TABLE 3:** Illustrative quotations from the expert interviews on stakeholder dialogues (Switzerland, 2024–2025).

ID	Theme	Quotation	Participant
Q1	1. Boundaries and core features	It’s a structured, democratic discussion… open but guided carefully, able to give voice to different perspectives	P11
Q2	1. Boundaries and core features	In a focus group you gather perspectives, not to decide; stakeholder dialogues are for actionable things: deciding or implementing a policy	P11
Q3	1. Boundaries and core features	A dialogue is a space where we bring together different individuals and organisations that have a stake… to develop potential solutions and recommendations, though many prefer to talk about options	P14
Q4	1. Boundaries and core features	Forcing consensus is inappropriate… one valid outcome is that there is no way forward	P02
Q5	1. Boundaries and core features	We respectfully listen to reach a joint position or, if not possible, at least agree on the differences and respect those	P10
Q6	2. Design levers – evidence inputs	A very good policy brief, well structured, not too long, is essential; otherwise it jeopardizes the dialogue	P06
Q7	2. Design levers – evidence inputs	Policy briefs bring everyone on the same page… based on high-quality evidence	P11
Q8	2. Design levers – agenda and moderation	The most crucial and time-consuming part is developing an agenda that takes us toward our goal and engages stakeholders	P10
Q9	2. Design levers – agenda and moderation	Moderation competence is very important; process specialists, not necessarily topic specialists, and with language competencies	P01
Q10	2. Design levers – agenda and moderation	Moderator skills: encourage receptivity, listen well, ensure clear language everyone understands, and make sure everyone speaks	P13
Q11	2. Design levers – format and methods	We always have breakout groups… people who will not speak in a big meeting get a chance to speak	P10
Q12	2. Design levers – format and methods	We ask stakeholders to build LEGO models for different scenarios, then weave them into a shared narrative where no model is left aside	P03
Q13	3. Power, equity, and legitimacy – stakeholder mapping	Stakeholders are selected with a matrix ‘Interests’ × ‘Influence’ for each intervention area, updated annually	P04
Q14	3. Power, equity, and legitimacy – stakeholder mapping	Use a list, then interest × impact… include those with agency for the decision and those affected who can become multipliers	P11
Q15	3. Power, equity, and legitimacy – representation	Very often stakeholder dialogues are elite-oriented; patient organisations are not legitimate representatives of all patients	P08
Q16	3. Power, equity, and legitimacy – representation	If the objective is a national solution and you do not have people from the French-speaking part, it’s not national	P01
Q17	3. Power, equity, and legitimacy – compensation	It’s worthwhile paying practitioners. They lose income; if not an honorarium, at least travel costs	P14
Q18	4. From talk to traction – early engagement	You should never surprise with your results. If stakeholders are involved, they’re not surprised; if not, they may be reluctant	P01
Q19	4. From talk to traction – commitments	At the conclusion… a two-to-four-page report summarising the outcome—Ms. X has committed to doing Y	P02
Q20	4. From talk to traction – follow-up mechanisms	We now run a modified Delphi—two rounds with a short online meeting in between to show results and build alignment	P14
Q21	5. What impact looks like	Success is a product that fulfils expectations… and that people go home with a good feeling	P01
Q22	5. What impact looks like	We run pre/post surveys, hold follow-up calls months later, and track citations and LinkedIn shares as soft indicators of uptake	P14
Q23	6. “Swissness” matters – policy preparation	We speak to those who actually do the policy preparation: insurers, doctors’ and nurses’ associations, rather than politicians	P14
Q24	6. “Swissness” matters – small-network dynamics	The country’s size fosters familiarity and trust, but the same individuals are frequently approached—overburdening key players—and a preference for consensus can slow discussions	P04
Q25	6. “Swissness” matters – small-network dynamics	Roundtable fatigue is real—too many, too costly, too little outcome	P08

### Boundaries and core features of stakeholder dialogues

Across interviews, participants differentiated stakeholder dialogue from adjacent participatory techniques (e.g., in-depth interviews, conventional focus groups). They consistently described it as a structured and democratic mode of deliberation anchored in synthesised evidence and oriented toward practical decisions or options for action. In this sense, dialogue is not primarily a data-gathering device but a decision-support mechanism that convenes actors with relevant knowledge and authority to act.

To make this concrete: participants described, for example, dialogues on the future of primary care and on strengthening the uptake of clinical guidelines in practice. In each case, the organising team prepared an evidence brief synthesising the available research and brought together stakeholders from public administration, professional associations, research, and practice, who worked in facilitated groups from a shared diagnosis of the problem toward options for action (P14). The stakeholders convened held the authority, resources, or experiential knowledge needed to act on the options discussed.

Participants emphasised that consensus is not a prerequisite for a successful dialogue. Its value lies in rendering the underlying reasoning public, specifying points of disagreement, and identifying the conditions under which movement is possible. Well-run dialogues may legitimately culminate in a clarified impasse, provided the reasons for that impasse are explicit and informative for subsequent work.

### Design levers that make (or break) deliberation

Stakeholder dialogues deliver more than talk when three mutually reinforcing levers are in place: (i) evidence inputs, (ii) agenda design and moderation, and (iii) format and methods. Each lever solves a specific problem in collective reasoning; together they create the conditions for fair, focused, and outcome-oriented deliberation.

#### Evidence inputs

Participants treated high-quality, accessible syntheses of evidence as the minimum condition for productive deliberation. Evidence briefs align starting assumptions and constrain drift.

Several organizing teams supplemented the main evidence brief with targeted preparatory materials for panellists (e.g., purpose statements, participant biographies, guiding questions, and clarification of the intended focus and boundaries of their contributions) to prevent unproductive divergence (P10).

#### Agenda design and moderation

The agenda was repeatedly identified as the primary design lever, and moderation as a professional skill emphasising process expertise over topic authority.

Operationally, facilitators reported summarising-and-checking moves (“this is what I heard: do we agree?”), explicit voting when required, and structured turn-taking to equalise participation (P07, P11).

#### Format and methods

Structural choices were used deliberately to distribute voice and dilute hierarchy effects. Breakout groups give quieter participants room; creative or embodied techniques externalise perspectives and foster shared construction.

Teams also used venue symbolism and interactional tone to shape contributions: alternative locations to signal creativity (P14), university settings to signal neutrality or seriousness (P01, P12), and informal forms of address to reduce social distance (P14). Online modes were used when necessary, typically with shorter sessions and tighter structure (P07, P11).

### Power, equity, and legitimacy

Who is in the room—and on what terms—was treated as central to fairness and impact. Most teams use a mapping frame (interest × influence or interest × impact) to balance decision-agency with lived experience.

Representation concerns were clearly identified: risks of elite orientation, weak legitimacy of some umbrella groups, and the “usual suspects” problem in small systems.

Participants reported practical equity tactics: delegated participation (community members elect representatives) to reduce stigma and burden (P12); gender-aware facilitation such as women-only breakout sessions to rebalance voice (P06); and compensation policies.

Handling conflicts of interest (COI) and role conflicts was framed as management rather than exclusion. Approaches varied from highly formal COI declarations and neutrality protocols (P10) to transparency without formal forms in knowledge-translation-oriented settings (P14). One recurrent move was to invite people as individuals (not as official representatives) to reduce positional bargaining, though some warned this could underplay institutional constraints downstream (P01).

### From talk to traction: products, commitments, and champions

Interviewees emphasised that a dialogue’s credibility rests on what it produces and what happens next. Outputs ranged from policy briefs, reduced core sets, and option lists to prototypes of policy strategies and visions (P07, P12, P14). Several stressed the importance of early engagement to prevent downstream resistance.

Commitments, named responsibilities, and follow-up were flagged as the bridge from words to action.

Follow-on mechanisms helped convert options into aligned positions. Running Delphi rounds after a dialogue to refine, prioritise, and maintain alignment was identified as particularly effective.

Others institutionalise monitoring of commitments or track whether dialogue options are referenced in subsequent planning (P02, P14). Some accepted that a credible outcome can be a clear “no way forward,” which prevents tokenistic consensus (P02).

### What impact looks like (and how it is evidenced)

Across cases, impact was described as slow, plural, and multi-trace. Near-term proxies included participant satisfaction and the production of a fit-for-purpose product; medium-term traces included pilots, policy mentions, or practice changes; soft indicators included citations, social shares, or follow-up calls.

Several interviewees cautioned against overspecifying impact claims when mandates are unclear; recommendations without implementers, budgets, or timelines risk stalling (P12). Others argued that documenting reasoning and clarifying conditions for movement are themselves valuable impacts in complex policy fields (P08).

### “Swissness” matters: system logics, languages, and scale

The Swiss context shaped design choices and expectations about uptake. First, many dialogues target policy-preparation actors (insurers, professional associations, cantonal officials) rather than elected politicians, given the political system’s built-in deliberation and the role of corporatist intermediaries.

Second, federalism and multilingual practice impose real constraints on selection, documentation, and facilitation. Materials and facilitation often need to be bilingual or multilingual; canton-level variation demands contextualisation; and inclusion of the French-speaking region is critical to legitimacy (P01, P07, P11, P12).

Third, small-network dynamics create both trust and fatigue:

Solutions included pragmatic tactics: piggybacking on existing events to reduce burden (P09, P11), pre-meeting onboarding so the main day can “go deep” (P14), and informality (venue and forms of address) to reset dynamics (P14). Language and accessibility were identified as aspects requiring active management (P10, P11, P12).

The three international interviews helped delineate what is specifically Swiss. Rather than describing a single shared model, they showed that how a dialogue is practised follows from the governance context of the organisation that initiates and hosts it. A participant at a global health organisation described large stakeholder consultations, governed by formal conflict-of-interest procedures, that fed directly into the organisation’s own work plans and the creation of a global advocacy alliance (P10). Another described “interest-holder” consultations for international guideline development, framed and ethically approved as research, noting that the term “stakeholder” itself had been abandoned in their setting at the request of Indigenous partners, who consider it colonial. A third emphasised dialogues embedded in community-based participatory research and interprofessional care teams (P13). Swiss participants, by contrast, consistently located dialogues in inter-institutional policy preparation among cantonal actors, insurers, and professional associations—several explicitly contrasting this with more centralised systems, where high-level policy dialogues convene decision-makers who are ready to act (P14).

## Discussion

This study contributes to the growing body of knowledge on stakeholder dialogues as mechanisms for translating research evidence into policy-relevant action. By eliciting the perspectives of experienced researchers and practitioners, our findings illuminate how dialogues are conceptualised, designed, and valued in practice, particularly within Switzerland’s pluralist, federal, and multilingual context. Three overarching insights emerge: stakeholder dialogues are distinctive not because they are participatory but because they constitute a deliberative infrastructure linking knowledge and action; their effectiveness depends less on procedural formalities than on the alignment of evidence, design, and facilitation; and contextual features—power relations, linguistic diversity, and scale—shape both the inclusiveness and legitimacy of deliberation. The interviews reaffirmed that stakeholder dialogues occupy a unique middle ground between technical analysis and political negotiation: a space where scientific evidence, experiential knowledge, and normative reasoning converge. This hybrid position echoes Lavis and colleagues’ [5] argument that evidence alone cannot determine policy choices; rather, structured deliberation enables actors to interpret evidence within their institutional and moral frameworks. Participants’ emphasis on reason-giving rather than consensus aligns with deliberative-democratic perspectives articulated by Gutmann and Thompson [9], in which legitimacy arises from transparent justification and mutual understanding rather than uniform agreement [9, 22]. Deliberative dialogues in Canadian mental health strategy development [13] similarly show how structured engagement with diverse actors can build trust and common ground on contentious issues.

Our findings extend Boyko et al.’s [1, 2] work positioning stakeholder dialogues as tools for clarifying policy options and fostering shared ownership. While earlier studies often emphasised tangible outputs such as lists of priorities, our participants highlighted processual impacts: clarification of disagreements, exposure of assumptions, and the generation of mutual respect. As Mitchell and colleagues [4] suggest, such epistemic and relational outcomes may be prerequisites for later coordination and implementation. Evaluating these effects requires attention to the quality of deliberation itself—whether participants engage with each other’s reasons, whether their understanding evolves, and whether trust or willingness to collaborate increases after the dialogue.

Three design levers—evidence inputs, agenda design and moderation, and format—shape the epistemic quality of deliberation. This triangulation resonates with Head’s [29] emphasis on combining credible evidence, inclusive deliberation, and pragmatic framing.

First, the strong emphasis on policy briefs confirms prior research on the knowledge-packaging challenge in evidence-informed policymaking [30, 31]. High-quality, balanced briefs function as boundary objects that align epistemic communities and establish a shared factual baseline, enabling participants to deliberate from common premises [32]. At the same time, preparing such briefs involves interpretive choices about evidence selection and framing, underscoring that evidence is never entirely neutral [6]. Future research should examine a wider range of evidence inputs, including rapid reviews, visual explainers, and digital evidence platforms.

Second, moderation emerged as a professional skill requiring neutrality, linguistic sensitivity, and process expertise. Particularly in multilingual and multidisciplinary settings, facilitators function as epistemic translators who bridge disciplinary idioms and ensure procedural fairness. The quality of deliberative exchanges depends heavily on the facilitator’s capacity to probe, synthesise, and guide discussion in real time.

Third, structural formats such as breakout groups, rotations, and collaborative exercises serve not only logistical but also normative purposes: they redistribute voice and mitigate hierarchy effects. This aligns with Fung’s [33] framework of empowered deliberation, which stresses that equality of participation must be intentionally designed. However, even well-designed formats cannot fully overcome deeper structural inequalities that shape participation and influence. Taken together, these elements form a pragmatic architecture for deliberation that sequences evidence, discussion, and synthesis.

A frequent criticism of participatory processes is that they generate deliberation without decision. Our findings offer a more nuanced view. Participants considered dialogues successful when they produced feasible commitments, clarified responsibilities, or identified conditions for progress. This pragmatic orientation aligns with action-oriented deliberation and realist evaluation perspectives [34], which emphasise mechanisms—such as shared understanding and early engagement—that enable later uptake in specific contexts.

Follow-up mechanisms, including Delphi rounds and structured reports, were cited as ways to sustain momentum and accountability. These hybrid approaches combine deliberation with decision tracking, supporting iterative evidence–policy interfaces. Importantly, some experts viewed transparent impasse as a legitimate outcome that clarifies barriers rather than masking disagreement, an interpretation consistent with adaptive governance principles [35].

Evaluating the impact of deliberative processes remains challenging. Participants described a continuum of impacts from mutual learning and satisfaction to policy reference and practice change, suggesting that traditional metrics may underestimate their value—a challenge compounded by a broader “crisis of democracy” that heightens both the need for and scepticism toward deliberative mechanisms [12]. They therefore combined “hard” indicators (policy uptake, implementation steps) with “soft” ones (follow-up engagement, diffusion of ideas), in line with knowledge-translation evaluation frameworks [24]. Recognising clarified disagreement as productive reframes impact as systemic learning rather than immediate consensus, echoing Bruen and Brugha [36] and Sienkiewicz and colleagues [15], who likewise located dialogues’ value in strengthened relationships and cross-sectoral understanding rather than immediate policy change.

Theoretically, these findings support viewing stakeholder dialogues as “boundary infrastructures” [37] connecting epistemic, institutional, and civic spheres—mechanisms at once for evidence translation, social learning, and collective reasoning. This threefold role situates dialogues at the core of what Parkhurst [6] calls the “good governance of evidence,” ensuring that knowledge informs policy effectively and legitimately.

Practically, the study suggests several design implications: integrating evidence and deliberation early through briefs and preparatory materials that align initial assumptions; investing in professional facilitation, since process expertise is as important as subject expertise; designing for equity through attention to power relations, compensation for participation, and formats that diversify voice; building follow-up mechanisms that turn dialogue into accountable commitments; and keeping dialogue models adaptable, as institutional, linguistic, and cultural conditions shape feasibility and legitimacy. These principles resonate with the design-thinking-for-policy movement [38], while grounding it more firmly in deliberative epistemology than in managerial efficiency.

### Limitations

As a qualitative study with a purposive expert sample, this research does not claim statistical generalisability. The perspectives gathered reflect experienced practitioners and may underrepresent community-level or political actors less embedded in evidence networks. Because the analysis focused on how experts design and value dialogues, we could not directly observe outcomes or trace long-term implementation paths. Policy briefs dominated our evidence-input data because participants most frequently mentioned them; future work should examine a wider range of inputs (e.g., rapid reviews, summaries, visual explainers, web-based evidence portals) and combine ethnographic observation with long-term policy tracking to understand how deliberative reasoning leads to institutional change. Comparative analyses across political cultures would further illuminate context–mechanism–outcome relationships.

### Conclusion

Stakeholder dialogues, when carefully designed and facilitated with deliberative integrity, are a powerful yet often underused tool for closing the gap between evidence and action in health policy. They shift the use of evidence from a transactional process to a relational one, emphasising that policy decisions are not just technical but also moral and communicative acts. In the Swiss context, where governance relies on negotiation among diverse actors and linguistic regions, dialogues exemplify the democratic value of inclusive reasoning. Beyond Switzerland, these insights support global efforts to make health policymaking more participatory, evidence-informed, and sensitive to context. Future efforts should focus less on increasing the number of dialogues and more on enhancing their quality, fairness, and follow-up, ensuring that deliberation moves beyond conversation to foster collective learning and coordinated action.
