# Supplementary material for: From evidence to action: expert perspectives on stakeholder dialogues in Swiss health policy
Source: Int J Public Health. 2026 Sep 18;71:1609991. doi: 10.3389/ijph.2026.1609991 (PMC13630709; doi:10.3389/ijph.2026.1609991)
Supplement: Supplementary file 1 [file DataSheet1.PDF]

Supplementary Material

Manuscript title: From evidence to action: expert perspectives on stakeholder dialogues in Swiss health policy

Supplementary Table S1. Interview domains, focus areas, indicative questions, and purpose; expert interview study on stakeholder dialogues in health policy, Switzerland and international, 2024–2025.

Domain	Main focus	Indicative questions	Purpose
**1. Understanding stakeholder dialogues**	Definitions, aims, and perceived value	"How do you define a stakeholder dialogue?" "Why are they important in your context?"	Explore conceptual boundaries and perceived roles of dialogues in linking evidence and decision-making.
**2. Experience and examples**	Professional contexts and applications	"Can you share examples of dialogues you have facilitated or attended?"	Capture diversity of experience and practical lessons across institutional settings.
**3. Types and formats**	Variation in structure, purpose, and participants	"Describe different types of dialogues you know." "How do objectives and participants vary?"	Identify typologies and contextual adaptations.
**4. Comparative methods**	Differences from focus groups or other participatory approaches	"How do stakeholder dialogues differ from focus groups or other qualitative methods?"	Clarify distinctive features and comparative advantages.
**5. Planning and organisation**	Preparation, stakeholder selection, logistics	"What steps are essential in planning?" "How do you identify and select participants?"	Document planning practices, logistical structures, and inclusion strategies.
**6. Roles and facilitation**	Responsibilities and conflict management	"What roles are essential in organising and facilitating?" "How are conflicts handled?"	Examine governance, facilitation skills, and conflict resolution mechanisms.
**7. Documentation and reporting**	Recording, summarising, and communicating outcomes	"How do you document and report dialogue proceedings?"	Understand mechanisms of transparency, accountability, and traceability.
**8. Outcomes and impact**	Effects, success indicators, and evaluation	"How do you assess success?" "What metrics are used?"	Elicit perceived criteria of effectiveness and evidence of policy influence.
**9. Challenges and facilitators**	Barriers, enablers, and best practices	"What are the common barriers and how are they overcome?"	Identify systemic and operational determinants of success.
**10. Contextual factors ("Swissness")**	Cultural and institutional influences	"Are there specific facilitators or challenges in Switzerland?"	Explore implications of federalism, multilingualism, and consensus culture.
**11. Guidance for practice**	Recommendations for a stakeholder dialogue handbook	"What sections or guidance should a handbook include?"	Generate transferable insights and structure for practical tools.

Supplementary Table S2. Completed Consolidated Criteria for Reporting Qualitative Research (COREQ) 32-item checklist

Reference: Tong A, Sainsbury P, Craig J. Consolidated criteria for reporting qualitative research (COREQ): a 32-item checklist for interviews and focus groups. Int J Qual Health Care (2007) 19(6):349–57.

No.	Item	Guide question / description	Response for this study
**Domain 1: Research team and reflexivity**			
**1**	Interviewer/facilitator	Which author(s) conducted the interviews?	Two members of the research team (initials withheld for blinded review) conducted all interviews.
**2**	Credentials	What were the researcher's credentials?	PhD-level researchers; one full professor and one post-doctoral researcher.
**3**	Occupation	What was their occupation at the time of the study?	Senior university academic and post-doctoral researcher in a Swiss university.
**4**	Gender	Was the researcher male or female?	Both female.
**5**	Experience and training	What experience or training did the researcher have?	Extensive experience in qualitative health research and stakeholder dialogue methodology; trained in qualitative methods.
**6**	Relationship established	Was a relationship established prior to study commencement?	Some participants were known to the research team through professional networks; two were also co-authors of the manuscript.
**7**	Participant knowledge of the interviewer	What did the participants know about the researcher?	Participants were informed of the study's aims, institutional affiliation, and the researchers' roles.
**8**	Interviewer characteristics	What characteristics were reported about the interviewer/facilitator?	The dual role of two co-authors as both participant and researcher was acknowledged; analytical safeguards are described in the Methods.
**Domain 2: Study design**			
**9**	Methodological orientation	What methodological orientation underpinned the study?	Reflexive thematic analysis (Braun & Clarke, 2019) with a hybrid deductive–inductive strategy.
**10**	Sampling	How were participants selected?	Purposive sampling, complemented by snowball sampling.
**11**	Method of approach	How were participants approached?	By email invitation.
**12**	Sample size	How many participants were in the study?	14 experts.
**13**	Non-participation	How many people refused to participate or dropped out?	Not systematically tracked; no drop-outs after consent.
**14**	Setting of data collection	Where was the data collected?	10 interviews via Zoom; 2 in person; 2 written responses.
**15**	Presence of non-participants	Was anyone else present besides the participant and researcher?	No.
**16**	Description of sample	What are the important characteristics of the sample?	See Table 1 of the main manuscript. Predominantly senior academics, research managers, and expert practitioners from Swiss and international health institutions.

No.	Item	Guide question / description	Response for this study
**17**	Interview guide	Were questions, prompts, guides provided by the authors?	Yes; see Supplementary Table S1. The guide was reviewed by the research team but was not pilot tested.
**18**	Repeat interviews	Were repeat interviews carried out?	No.
**19**	Audio/visual recording	Did the research use audio or visual recording?	Yes; audio recording for all verbal interviews.
**20**	Field notes	Were field notes made during and/or after the interviews?	Yes; simultaneous field notes during each interview.
**21**	Duration	What was the duration of the interviews?	30–60 minutes.
**22**	Data saturation	Was data saturation discussed?	Yes; thematic sufficiency was monitored and is discussed in the Methods section.
**23**	Transcripts returned	Were transcripts returned to participants for comment?	No.
**Domain 3: Analysis and findings**			
**24**	Number of data coders	How many data coders coded the data?	Two initial coders; four authors consolidated the codebook.
**25**	Description of the coding tree	Did authors provide a description of the coding tree?	A codebook was developed iteratively; a thematic overview is presented in Table 2 of the main manuscript.
**26**	Derivation of themes	Were themes identified in advance or derived from the data?	Hybrid approach: sensitising domains from the interview guide (deductive) combined with emergent coding (inductive).
**27**	Software	What software was used to manage the data?	Not specified; manual coding.
**28**	Participant checking	Did participants provide feedback on the findings?	No formal member checking was conducted.
**29**	Quotations presented	Were participant quotations presented to illustrate themes?	Yes; quotations are identified by participant ID (P01–P14).
**30**	Data and findings consistent	Was there consistency between the data presented and the findings?	Yes; themes are supported by multiple quotations across participants.
**31**	Clarity of major themes	Were major themes clearly presented?	Yes; six major themes with subthemes are presented in Table 2 and elaborated in the Results.
**32**	Clarity of minor themes	Is there a description of diverse cases or minor themes?	Minor variations (e.g., differences between Swiss and international perspectives, online versus in-person formats) are discussed within each theme.
